# Modified Extracorporeal Photopheresis with Cells from a Healthy Donor for Acute Graft-versus-Host Disease in a Mouse Model

**DOI:** 10.1371/journal.pone.0105896

**Published:** 2014-08-22

**Authors:** Holger Budde, Susanne Kolb, Laura Salinas Tejedor, Gerald Wulf, Holger M. Reichardt, Joachim Riggert, Tobias J. Legler

**Affiliations:** 1 Department of Transfusion Medicine, University Medical Center Göttingen, Göttingen, Germany; 2 Department of Hematology and Oncology, University Medical Center Göttingen, Göttingen, Germany; 3 Institute for Cellular and Molecular Immunology, University Medical Center Göttingen, Göttingen, Germany; Beth Israel Deaconess Medical Center, Harvard Medical School, United States of America

## Abstract

**Background:**

Graft-versus-host disease (GvHD) is a major challenge after hematopoietic stem cell transplantation but treatment options for patients are still limited. In many cases first-line treatment with glucocorticoids is not successful. Among second-line therapies the extracorporeal photopheresis (ECP) is frequently performed, due to induction of selective tolerance instead of general immunosuppression. However, for some patients with severe acute GvHD the leukapheresis step of the ECP procedure is physically exhausting and limits the number of ECP cycles.

**Methods:**

We hypothesized that leukocytes from healthy cell donors could be used as a replacement for ECP leukocytes gained from the GvHD patient. For this purpose we used a well established mouse model of acute GvHD. The ECP therapy was based on cells with the genetic background of the initial donor of the stem cell transplantation. As a precondition we developed a protocol representing conventional ECP in mice equivalent to clinical used ECP setup.

**Results:**

We could demonstrate that conventional, clinically derived ECP setup is able to alleviate acute GvHD. By using leukocytes obtained from healthy mice with the bone marrow donor’s genetic background we could not observe a statistically significant therapeutic effect.

**Conclusions:**

Conventional human ECP setup is effective in the mouse model of severe acute GvHD. In addition we could not prove that ECP cells from healthy mice with bone marrow donor’s genetic background are as effective as ECP cells derived from GvHD mice. Based on our findings, new questions arise for further studies, in which the cellular characteristics for ECP mediated immune tolerance are a matter of investigation.

## Introduction

Patients with malignant diseases of the hematopoietic system such as leukemia still have limited therapeutic options. Allogeneic hematopoietic stem cell transplantation (HSCT) is often performed to obtain a long-term disease-free survival [1]. However, HSCT is associated with severe side effects, of which some can be fatal. The graft-versus-host disease (GvHD) belongs to these side effects and is one of the major limiting factors in hematopoietic stem cell transplantation [2]. In brief, GvHD is based on alloreactive T-cells from the stem cell graft, which attack several organs of the recipient and cause a generalized tissue rejection [3]. Especially acute GvHD is associated with high morbidity and mortality [4]. First-line therapy of acute GvHD is mainly based on glucocorticoids [5]. However, after HSCT patients often suffer from reduced immunocompetence due to the myeloablative conditioning regimen. The use of glucocorticoids in these cases increases the risk of opportunistic infections. Furthermore, in steroid-resistant or steroid-refractory patients there is a need for alternative therapeutic interventions [6]–[8].

Extracorporeal photopheresis (ECP) is a widespread used second-line treatment for GvHD. It is frequently used for chronic GvHD but also effective for the treatment of acute GvHD with response rates up to 80% depending on the affected organ [9], [10]. In comparison to other immunosuppressive therapies, ECP is rarely associated with side effects [11]. One major advantage of ECP therapy is the induction of immunotolerance without general immunosuppression. The frequency of opportunistic infections is not increased by the ECP [12], [13]. Due to its efficacy and the low risk of side-effects, some groups suggest using ECP as part of the first-line therapy for acute GvHD [14]. During the ECP procedure, patient’s own leukocytes are isolated by apheresis. The cells are treated *ex vivo* with 8-Methoxypsoralen (8-MOP) and UV-A light, a process, which causes cellular apoptosis [15]. Subsequently, the cells are transfused to the patient. Although up to 10% of the peripheral T-cells are eliminated by a single ECP procedure, the reduction of allo-reactive T-cells is probably not the key mechanism of ECP. More likely, 8-MOP/UV-A treated apoptotic cells are phagocytized and processed by antigen-presenting cells, a mechanism which initiates immune tolerance [16]. An increased number of regulatory T-cells (Tregs) was frequently observed after ECP therapy and is supposed to be a major cause of ECP mediated immune tolerance [17].

However, not all GvHD patients respond to ECP therapy and especially for patients suffering from severe acute GvHD the apheresis procedure can be a physical burden. In particular for these patients, modifications of the ECP procedure could be beneficial in the context of ECP application comfort. By replacing patient’s leukocytes by the one’s from blood donors, the patient apheresis would be omitted and ECP could be applied more frequently even to patients with severe GvHD.

Considering this, the aim of our study was to investigate whether 8-MOP/UV-A treated leukocytes from the donor of the original stem cell graft are able to alleviate severe acute GvHD *in vivo*. Therefore, we used a well known mouse model of acute GvHD and established a protocol for a standard murine ECP therapy, which is comparable to conventional human clinical ECP protocols. The therapeutic cells were taken from mice with GvHD after bone marrow transplantation, which had the same genetic background as the ECP treated mice. By using this protocol we could show for the first time that conventional ECP leads to reduced mortality rates in a MHC mismatched mouse model of severe acute GvHD. In a second step we used a modified ECP protocol, in which cells from the untreated bone marrow donor strain were used instead of cells from transplanted GvHD mice.

Altogether, the goal of our study was to examine whether a modified ECP setup with cells derived from healthy non-GvHD mice is able to improve the course of acute GvHD. Although we could not prove that such a modified ECP setup is therapeutically effective, our observations are important for the investigation of further modifications to improve the efficacy of the ECP therapy.

## Methods

### Mice

Male BALB/c recipient mice were purchased from Charles River Laboratories (Sulzfeld, Germany). Male C57BL/6J (B6) donor mice were purchased from Janvier (St Berthevin Cedex, France). Mice were between 10 and 12 weeks of age when BMT was performed. Animals were housed under pathogen-free conditions in individual ventilated cages.

### Ethics statement

All animal experiments were conducted according ethical regulations and approved by the corresponding authorities (Niedersächisches Landesamt für Verbraucherschutz und Lebensmittelsicherheit, permit number 33.9-42502-04-11/0472). Animals were treated with metamizole analgesics and comprehensive efforts were performed to minimize suffering. For further details about the animal experiments see Table S1 in the supporting information.

### Bone marrow transplantation

Resembling human HSCT, allogeneic bone marrow transplantation (BMT) was performed in mice for induction of acute GvHD (modified after Tischner et al., [18]). Conditioning regimen consisted of a single total body irradiation (TBI) with a dose of 9.5 Gy. Intravenous transfusion of 10^7^ syngeneic or allogeneic bone marrow (BM) cells and 2×10^6^ T-cells were performed 24 h after TBI. BM cells were prepared from femur and tibia and T-cells were depleted from BM with CD90.2 MicroBeads (Miltenyi Biotec, Bergisch Gladbach, Germany). T-cells were isolated from spleen and lymph nodes by magnetic beads (Pan T-cell Isolation Kit, Miltenyi Biotec). To avoid bacterial infection, neomycin was added to the drinking water.

### Chimerism analysis

Molecular chimerism was assessed 8 weeks after T-cell depleted BMT. CD45^+^/CD229.1^+^ cells were counted as BALB/c leukocytes and CD45^+^/CD229.1^−^ cells as leukocytes with B6 background [19].

### GvHD pathology analysis

Animals were scored daily for GvHD symptoms. The disease score consisted of 4 criteria including posture, activity, fur/skin and diarrhea with a maximum of 2 points each. Maximum score was 8 but animals reaching a score of 6 due to disease severity were sacrificed. Animal weight was assessed daily.

### ECP

Splenocytes were prepared from a second cohort of B6→BALB/c transplanted animals after showing first clinical signs as a model for “conventional ECP” or from healthy untreated B6 animals as a model for the “modified ECP”. After red blood cell lysis, cells were incubated with 8-MOP (0.2 µg/ml) for 30 min prior to UV-A irradiation (2 J/cm^2^). Afterwards cells were washed and diluted in PBS for intravenous tail vein injection. ECP treatment was performed weekly for three times for conventional ECP and, due to the lack of significant therapeutic response, four times for modified ECP. T-cell dose was 1×10^6^ cells for conventional ECP and the modified ECP was performed with 1×10^6^ and 5×10^6^ cells in two different series, respectively. Control animals were injected with PBS only. In every experimental series, an own PBS control group was carried in parallel for precise analysis excluding shifts in GvHD score severity due to performing therapy and control series at different time points. Additionally, in each experimental series ECP treated animals and PBS control mice were housed together. Each experiment was performed in at least two independent series.

### Flow cytometric analysis

Cells were analyzed when animals reached a disease score of 6 or at day 56 after BMT if score 6 was not reached before. Fc receptors were blocked with CD16/32 Fc receptor block (eBioscience, San Diego, CA, USA). Anti-mouse CD3e (Pacific Blue, clone 145-2C11, Armenian Hamster IgG) was purchased from BioLegend (San Diego, CA, USA). CD4 (FITC, clone RM4-5, Rat IgG2a) and CD45 (FITC, clone 30-F11, Rat IgG2b) were purchased from eBioscience and CD229.1 (APC, clone 30C7, IgG2a) from BD Bioscience-Pharmingen (San Diego, CA, USA). Cells were incubated with antibodies for 20 min at 4°C, washed with PBS and analysed by flow cytometry using a FACS Canto II flow cytometer (BD Biosciences, San Jose, CA, USA). Mouse Regulatory T-cell Staining Kit 2 was purchased from eBioscience and used according to the manufacturer’s recommendations.

### Statistical analysis

Survival rates were compared by using Kaplan-Meier plots and log-rank test was used for calculation of the corresponding p-values. Flow cytometry data from different groups were compared by using the non-parametric Mann-Whitney U test. All calculations were performed with “STATISTICA Version 10” software (StatSoft Inc., Tulsa, USA). Graphs are shown as bars with mean ± SD or, in case of high variance, as dot-plots with median.

## Results

### Allograft T-cell depletion completely prevents GvHD in MHC mismatched mouse model

**Figure 1:**
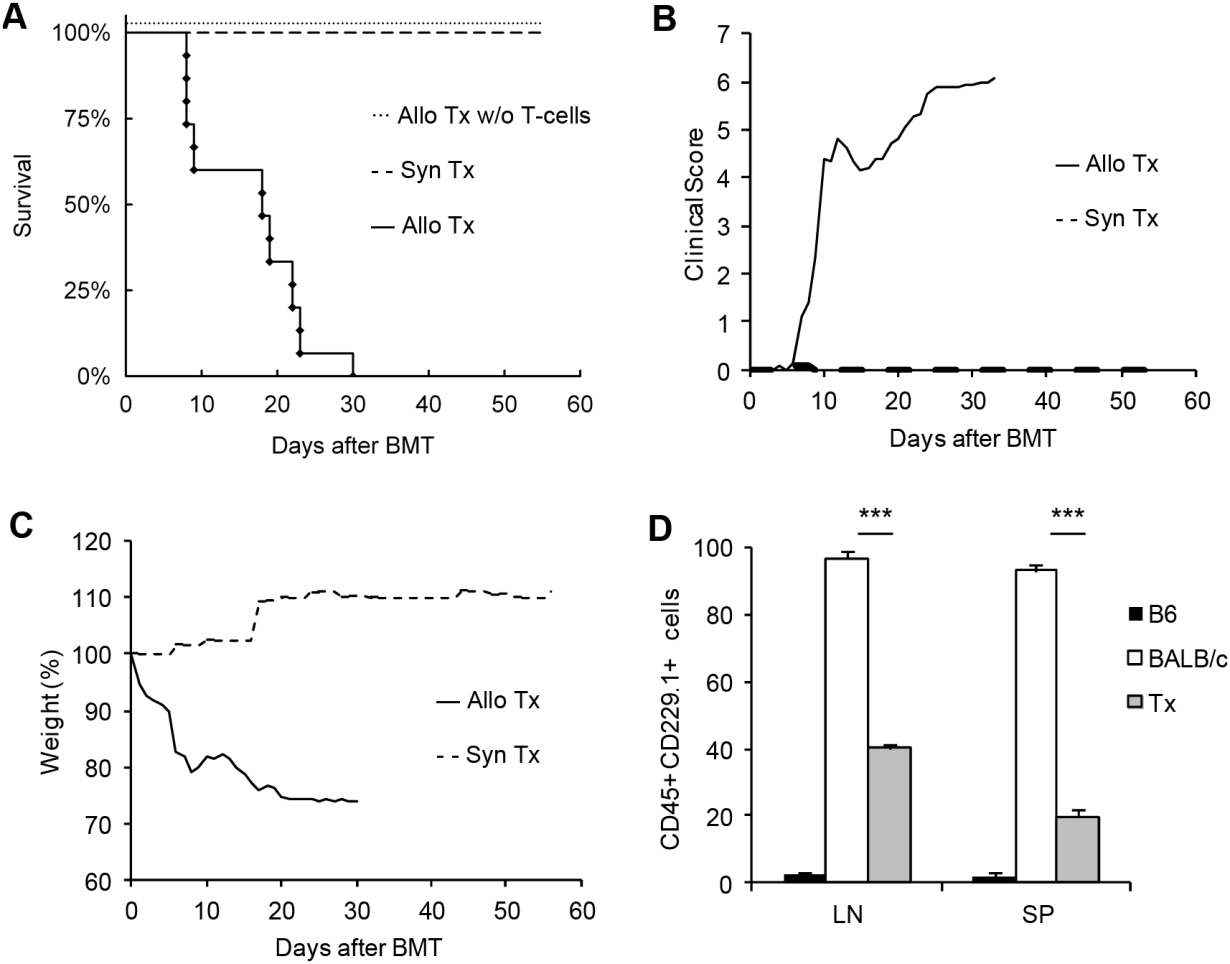
Major histocompatibility complex (MHC) mismatched mouse model of allogeneic BMT for GvHD induction. Survival after allogeneic BMT in a C57BL/6 (B6)→BALB/c mouse model (n = 15) of acute GVHD (**—**♦) (A). Both, syngeneic BALB/c→BALB/c BMT (n = 11) and allogeneic T-cell depleted B6→BALB/c BMT (n = 3) were performed as transplantation controls. Both control groups are represented as dashed lines with a survival rate of 100%. Daily assessed clinical score of allogeneic (including T-cells) vs. syngeneic transplanted mice (B). Weight changes in allogeneic (including T-cells) vs. syngeneic transplanted mice (C). Assessment of engraftment by cellular chimerism analysis 56 days after BMT in the T-cell depleted B6→BALB/c mouse model (D). CD45 positive leukocytes were stained for expression of CD229.1 antigen which is expressed on BALB/c leukocytes but not on B6 cells. Percentages of CD45^+^ CD229.1^+^ leukocytes from all splenocytes (SP) or lymph node cells (LN) are shown. Stainings were performed for wild-type B6 (black bars), wild-type BALB/c (white bars) and B6→BALB/c transplanted mice (Tx, grey bars). Chimerism analysis was performed in 3 animals from each group (mean ± SD, ***p<0.001).

We used a well known mouse model of acute GvHD with B6 (H2^b^) as donor and BALB/c (H2^d^) as recipient mouse strain. The model is based on MHC I and MHC II mismatches and GvHD is mediated by both CD4^+^ and CD8^+^ T-cells [20]. Due to the reproducible and robust development of GvHD symptoms, it is one of the most studied mouse models for acute GvHD [20], [21]. In most cases, allogeneic transplanted mice did not survive the experimental observation time of 56 days (Fig. 1A). Animals showed clinical symptoms such as diarrhea, rough fur, hunchback posture and reduced activity accompanied by weight loss (Fig. 1B and C). GvHD outcome and lethality was most distinct after the first and after the third week, respectively. In contrast, all control animals after syngeneic BALB/c→BALB/c transplantation survived without any symptoms (Fig. 1A–C). Furthermore, BALB/c mice transplanted with allogeneic T-cell depleted B6 bone marrow without T-cells from spleen or lymph node did not develop any disease symptoms (Fig. 1A), highlighting the pivotal role for allogeneic T-cells in GvHD induction. To control successful engraftment, we analyzed the chimerism level after T-cell depleted BMT. For this purpose we used a CD229.1 antibody, which reacts with BALB/c leukocytes but not with cells from the B6 strain. At day 56 after BMT we found ∼40% of CD45^+^ CD229.1^+^ cells in lymph nodes and ∼20% in spleen which corresponds to B6 donor leukocyte background in transplanted BALB/c mice of about 60% in lymph nodes and 80% in spleen (Fig. 1D).

### GvHD is linked to reduced regulatory T-cell level

**Figure 2:**
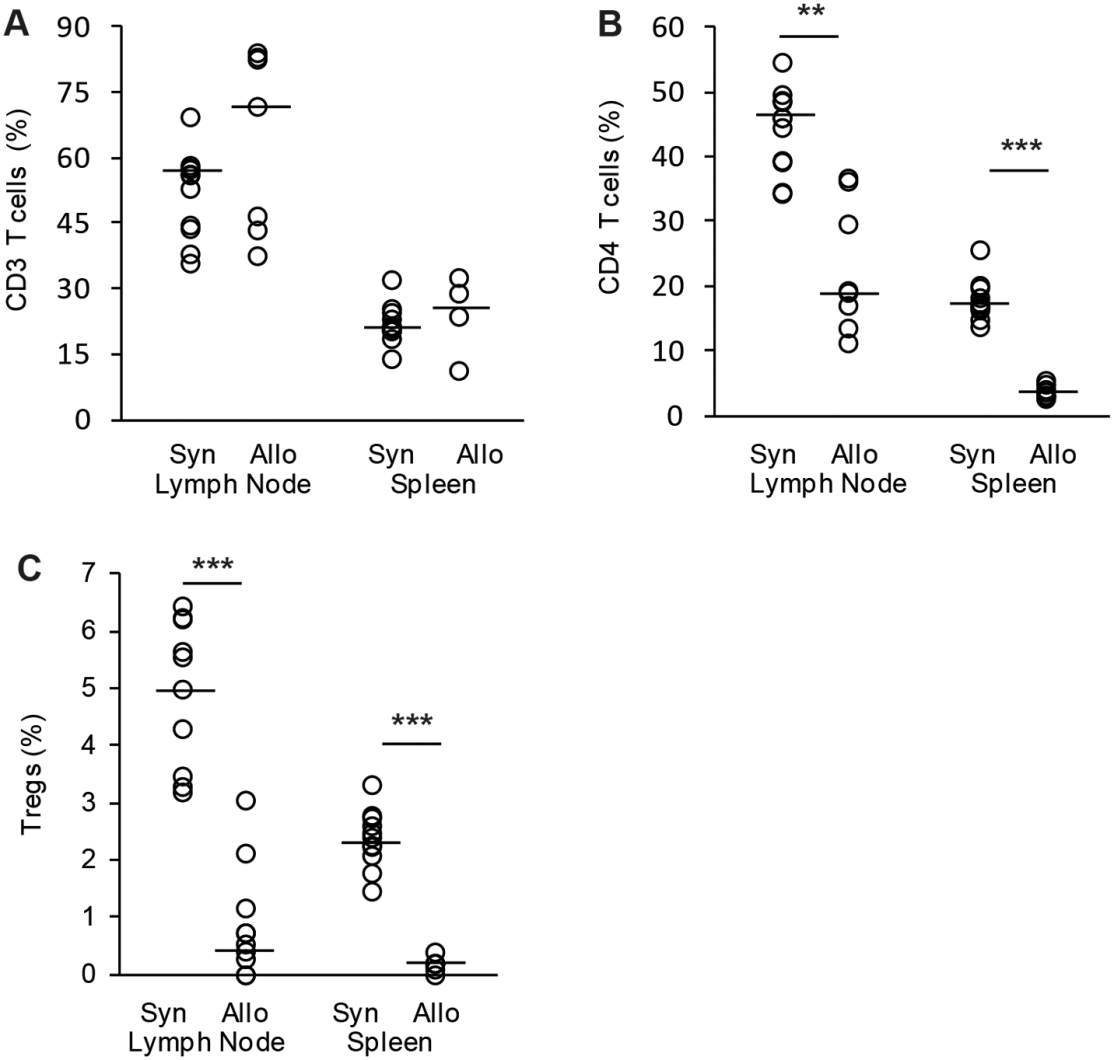
Evaluation of T-cell populations after syngeneic and allogeneic BMT. Analysis of T-cell subpopulations were performed after BMT in allogeneic C57BL/6→BALB/c (Allo) vs. syngeneic control transplantations (Syn). Percentage of CD3 positive T-cells (A), CD4 positive T-helper cells (B) and CD4 CD25 FoxP3 positive regulatory T-cells (C) from all splenocytes or lymph node cells were shown. Cells were analyzed from 7 allogeneic and 11 syngeneic transplanted mice. Animals were sacrificed when reaching clinical GvHD score ≥6 or when reaching day 56 after BMT (dot-plot with median, **p<0.01, ***p<0.001).

Since T-cells are key players in GvHD induction, we looked for different T-cell levels in lymph nodes and spleen after BMT. Allogeneic transplanted mice were analyzed at individual experimental break-off when reaching GvHD clinical score of 6 and syngeneic transplanted animals were analyzed 56 days after BMT. There was no difference in overall CD3 T-cell percentage between allogeneic transplanted animals suffering from GvHD compared to healthy syngeneic transplanted mice (Fig. 2A). In contrast, CD4 T-helper cell percentages of GvHD animals were significantly reduced in both lymph nodes and spleen (Fig. 2B). Furthermore, we could observe a massive reduction of CD4^+^ CD25^+^ FoxP3^+^ regulatory T-cells (Tregs) in GvHD animals (Fig. 2C).

### Conventional ECP ameliorates acute GvHD in a murine MHC major mismatched model

**Figure 3:**
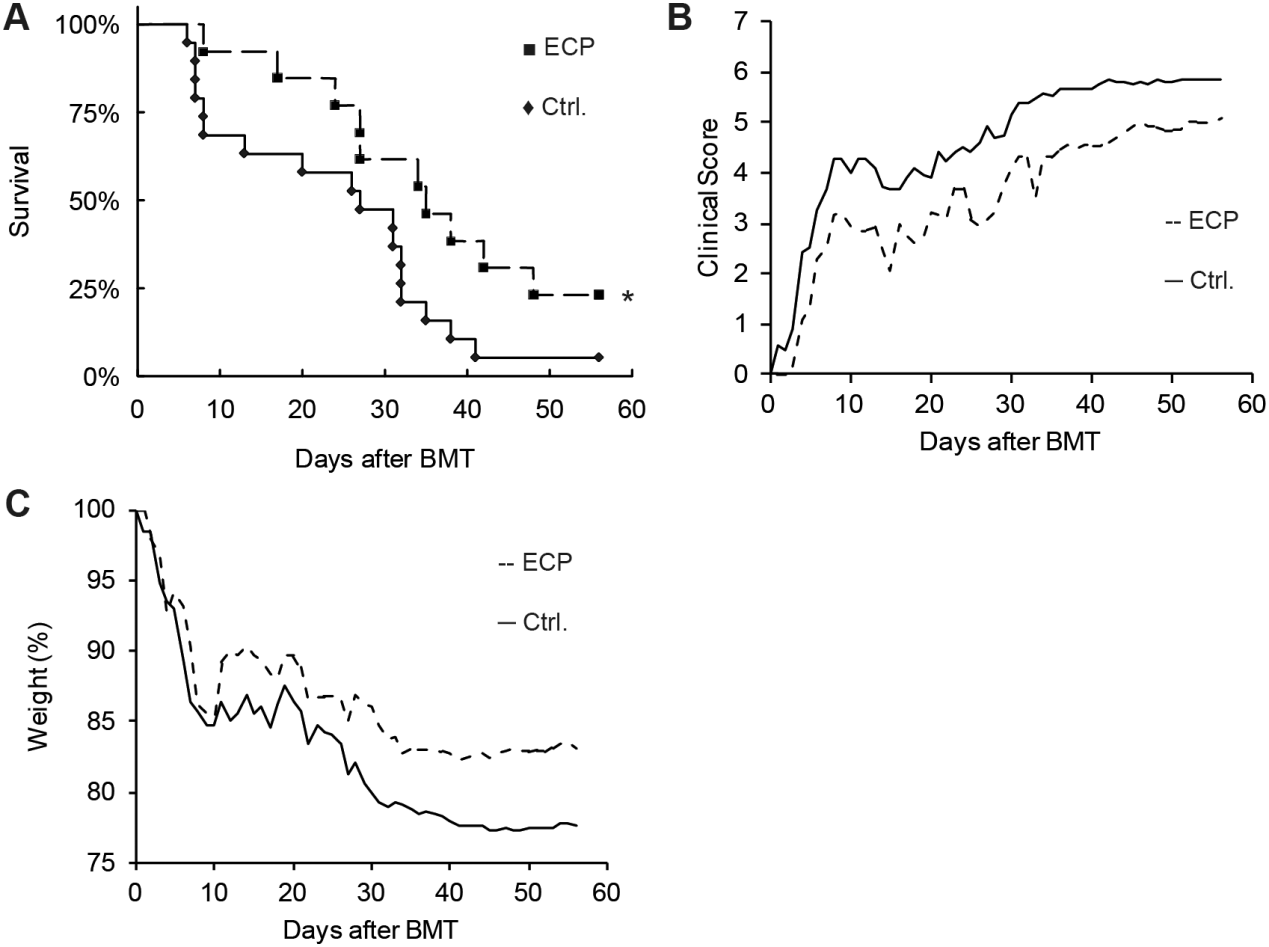
Conventional ECP therapy in the mouse model of acute GvHD. The therapeutic impact of conventional extracorporeal photopheresis (ECP) on GvHD severity in the C57BL/6→BALB/c mouse model was assessed. Animals of the ECP group (**---**▪) were injected with 8-Methoxypsoralen and UV-A treated splenocytes from another cohort of C57BL/6→BALB/c transplanted GvHD animals with identical genetic chimerism. Animals received 3 ECP treatments in weekly intervals, started at day 1 after BMT. Control animals (**—**♦) received PBS injections at the same time points. Kaplan-Meier plot showing survival rates of the ECP treated vs. PBS control group (A). The ECP group consisted of 13 and the control group of 19 animals (*p<0.05, log rank test). Clinical GvHD score (B) and weight loss (C) were assessed daily.

**Figure 4:**
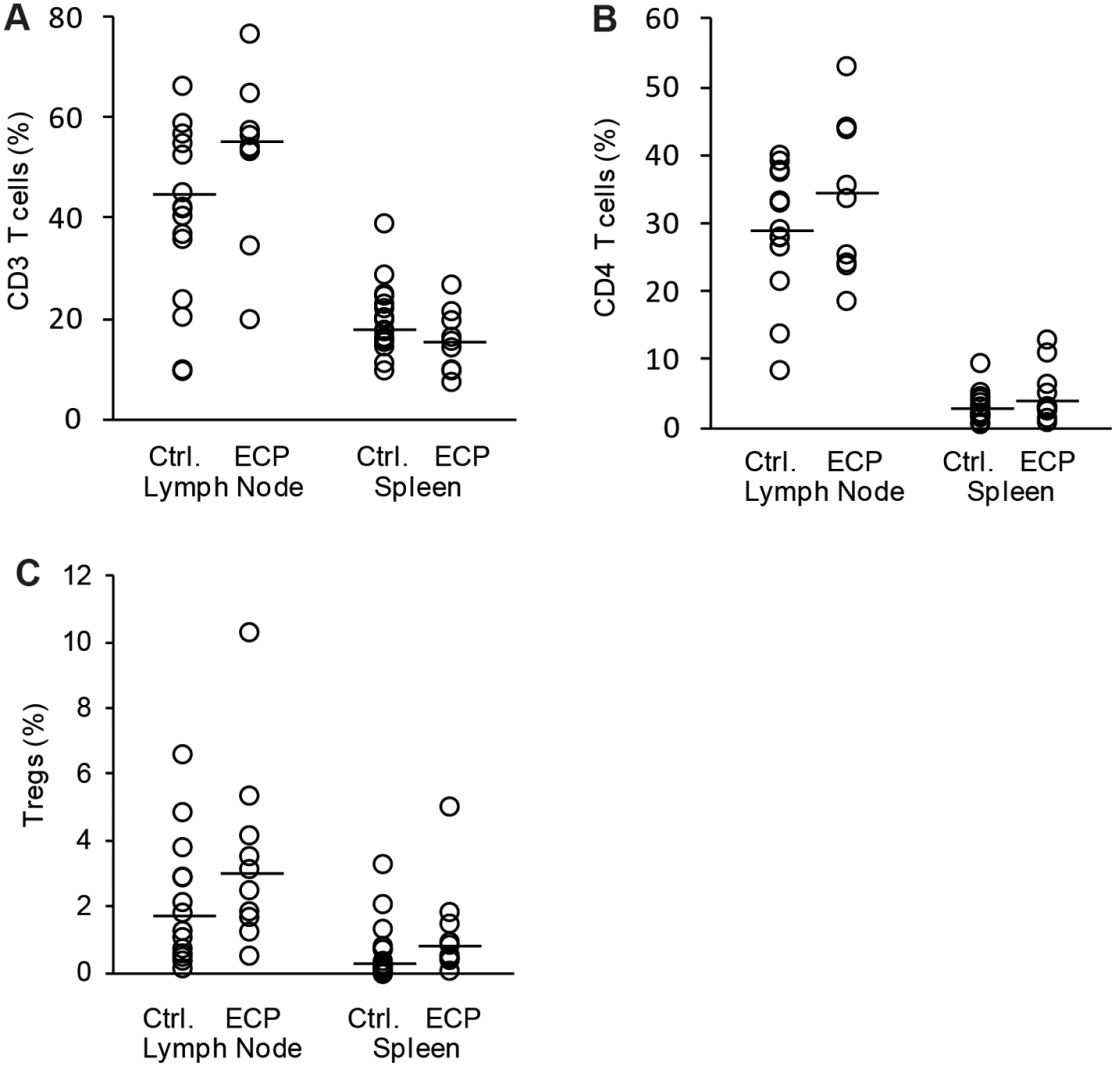
Analysis of T-cell subpopulations in GvDH mice treated with conventional ECP. GvHD was induced by allogeneic C57BL/6→BALB/c BMT and ECP treatment was performed weekly starting at day 1 after BMT (ECP). GvHD induction in control animals (Ctrl.) was performed simultaneously and animals received weekly PBS injections instead of ECP treatments. Percentage of CD3 positive T-cells (A), CD4 positive T helper cells (B) and CD4 CD25 FoxP3 positive Tregs (C) from all lymph node cells or splenocytes. Analysis is based on 10 ECP treated and 14 control mice. Animals were sacrificed when reaching clinical GvHD score ≥6 or at day 56 after BMT (dotplot with median).

ECP is a widespread used clinical therapy for GvHD. We transferred the clinical used human ECP setup in our mouse model. Splenocytes from another cohort of allogeneic transplanted B6→BALB/c mice with GvHD symptoms were prepared and treated with 8-MOP and UV-A irradiation. Following 8-MOP/UV-A treatment, 1×10^6^ splenocytes were injected intravenously in ECP recipient mice. The number of 1×10^6^ splenocytes is about 10% of the mouse’s peripheral leukocytes. This corresponds to the clinical ECP procedure in which about 10% of the peripheral leukocytes are treated *ex vivo* after apheresis [22]. Our murine ECP therapy was initiated one day after BMT and performed three times at weekly intervals. Compared to the control group injected with PBS only (n = 19), ECP treated animals (n = 13) had a significantly higher survival rate, demonstrated by Kaplan-Meier curves and log-rank test (p<0.05, Fig. 3A). Two months after BMT, three (23%) ECP treated animals were still alive, whereas only one (5%) control animal survived. Notably, during the first GvHD wave which occurs in between day 7 and 10 after BMT, survival was markedly reduced in the control group. In the ECP group only 1 of 13 (8%) animals did not survive the first GvHD wave compared to 6 of 19 (32%) animals in the control group. During the observation time of 56 days, the average clinical score of the ECP treated animals was around 1 point less than of the control animals (Fig. 3B). Furthermore, control animals had slightly but not significant higher final average weight loss (−22.4%) than animals from ECP treatment group (−16.9%, Fig. 3C). Concerning T-cell percentages of all splenocytes or lymph node cells, we could not find significant differences in CD3^+^ cytotoxic T-cells, CD4^+^ T-helper cells or Treg fractions between control and ECP treated animals (Fig. 4A–C). In comparison to the positive therapeutic effects described above, there was no effect on GvHD outcome and survival rates when performing just one single ECP treatment only (data not shown), suggesting that repeated ECP treatments are required for a therapeutic effect.

### Modified ECP setup is less effective than conventional ECP

**Figure 5:**
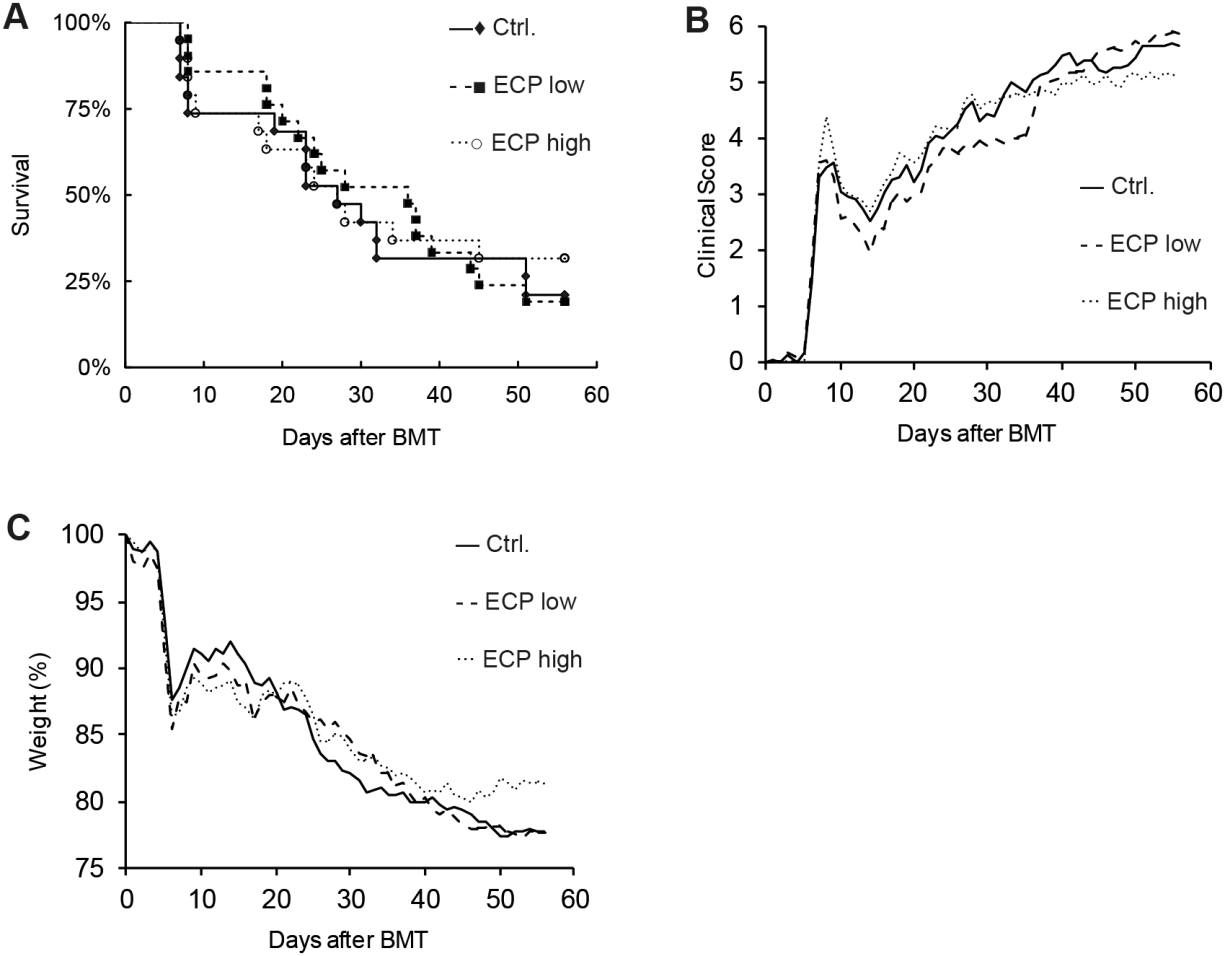
Modified ECP therapy for acute GvHD. Modified ECP therapy was performed as treatment for acute GvHD induced by C57BL/6→BALB/c bone marrow transplantation. ECP cells were isolated from wild type B6 spleens and treated with 8-Methoxypsoralen (8-MOP) and UV-A light. Treated cells were injected 4 times in C57BL/6→BALB/c transplanted GvHD mice at weekly intervals starting from day 1 after BMT. For each therapeutic injection, 8-MOP/UV-A treated cells were used either in low concentration of 1×10^6^ cells (**---**▪) or in high concentration of 5×10^6^ (···○) cells. Control animals (**—**♦) were transplanted simultaneously and injected with PBS only at therapeutic time points. Therapeutic impact and GvHD severity were analyzed by comparing survival rates (A), clinical GvHD symptom score (B) and weight loss (C).

**Figure 6:**
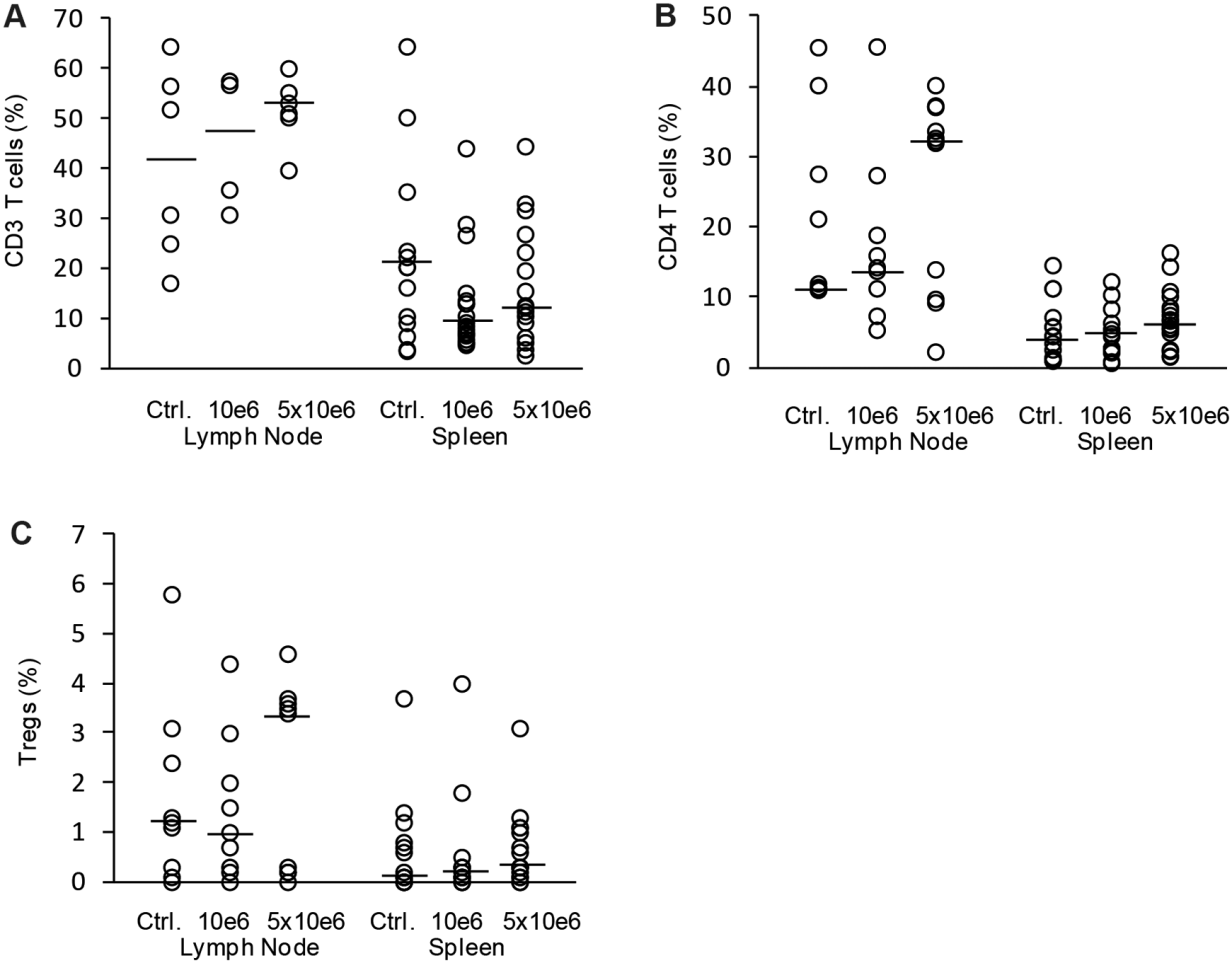
Assessment of T-cell populations after modified ECP therapy. T-cell subpopulations in GvHD mice treated by modified ECP were analyzed. ECP cells were isolated from wild type C57BL/6 spleens and treated with 8-Methoxypsoralen and UV-A light. Subsequently splenocytes were injected in GvHD mice after allogeneic B6→BALB/c bone marrow transplantation. ECP was performed 4 times at weekly intervals with cell concentrations of either 1×10^6^ cells or 5×10^6^ cells. Control animals were treated with PBS in parallel (Ctrl.). Percentage of CD3 positive T-cells (A), of CD4 positive T-helper cells (B) and CD4 CD25 FoxP3 positive Tregs (C) from all lymph node cells or splenocytes. Analysis is based on 9 animals treated with 1×10^6^ cells (10e6), 11 animals treated with 5×10^6^ cells (5×10e6) and 9 control mice (Ctrl.). Animals were sacrificed when reaching clinical GVHD score ≥6 or at day 56 after BMT (dot-plot with median).

Conventional ECP is based on patient’s own leukocytes, which are treated *ex vivo* with 8-MOP/UV-A and subsequently retransfused to the patient. A common clinical setup for the treatment of acute GvHD consists of two ECP procedures on consecutive days followed by further treatments at different intervals. However, a high frequency of apheresis cycles is not possible in all patients with severe GvHD. To maintain ECP frequency and apoptosis level in these patients, other cell sources for 8-MOP/UV-A treatment would be a major improvement for ECP application. The most standardized cell source would consist of allogeneic leukocytes of the healthy stem cell donor of the preceding stem cell transplantation. In addition, in case of previous related stem cell transplantation, donors would probably be well motivated for further leukocyte donations. To test whether mouse leukocytes with genetic background of the BMT donor are functional in ECP therapy, we used splenocytes from healthy B6 mice for 8-MOP/UV-A treatment and subsequent injection into B6→BALB/c transplanted GvHD mice. As well as for conventional ECP, we used 1×10^6^ 8-MOP/UV-A treated splenocytes at weekly intervals. In the modified ECP we observed a slight but not significant improvement in the survival rate at least within the first 35 days after BMT (Fig. 5A). The clinical score was similarly reduced but there was no difference in body weight loss between control and ECP group despite of an additional fourth ECP cycle (Fig. 5B and C). To analyze, whether more cells are required in the modified ECP setup, we also tested the therapeutic effect of 5×10^6^ 8-MOP/UV-A treated B6 splenocytes in B6→BALB/c transplanted mice. In this treatment group four ECP cycles were performed at weekly intervals. On the one hand, increasing the therapeutic cell concentration to 5×10^6^ cells did not result in improved clinical score, increased body weight or higher survival rate (Fig. 5A–C). On the other hand, we could not observe any negative or alloreactive effect using increased numbers of B6 splenocytes, suggesting that B6 T-cells were sufficiently inactivated by 8-MOP/UV-A treatment. Accordingly, cellular analysis of splenocytes and lymph node cells showed no difference in CD3^+^ T-cell, CD4^+^ T-cell or CD4^+^ CD25^+^ FoxP3^+^ Treg percentages between therapy and control groups (Fig. 6A–C).

## Discussion

Appropriate *in vivo* models are essential for the development of new GvHD therapies. In order to investigate the impact of ECP on severe acute GvHD, we chose the B6→BALB/c full MHC mismatched model. By using this model we observed severe GvHD signs and poor survival of untreated control mice, which is common for this model [23]. In contrast, T-cell depleted BMT does not lead to any GvHD symptoms due to the lack of alloreactive T-cells from the B6 donor strain, an observation which proves the validity of our model. These findings are in concordance with less frequent GvHD after T-cell depleted allogeneic HSCT in humans [24]. Additionally, we could proof by chimerism analysis of T-cell depleted animals that engraftment occurred in our BMT mouse model. For all subsequent therapeutic ECP experiments we performed transplantations with bone marrow and donor T-cells. Since T-cells are important for improved engraftment in allogeneic transplant settings, the chimerism of GvHD mice transplanted with both bone marrow and T-cells should be even higher towards donor cell population.

After having set up the *in vivo* model of acute GvHD we established a model of treating mice with ECP previously described by Gatza et al. [25]. In this model, cells for ECP were taken out of a second cohort of GvHD mice after allogeneic BMT to treat the first cohort of GvHD mice. Gatza and co-workers found that conventional ECP had a therapeutic effect in a MHC matched but minor histocompatibility mismatched mouse model of GvHD. Looking for the impact of ECP in the well known full MHC I and II mismatched B6→BALB/c model, we could demonstrate for the first time in mice that this conventional ECP setup is also effective in acute GvHD after BMT. In contrast to the work of Gatza et al. [25] and the findings in the contact hypersensitivity mouse model of Maeda et al. [26], we could not find increased Treg levels in mice treated with ECP. However, many of our ECP treated animals survived the observation time of 56 days. Hence, we analyzed cellular samples four weeks after the last ECP procedure. Thus, it is possible that Treg levels were higher directly after ECP but not after the full observation time. Further studies on the kinetics of Treg after ECP treatment could reveal new information for the development of the best ECP schedule.

From clinical experience it is well known that frequent repetition of the ECP procedure is required for a positive therapeutic outcome [27]. In our mouse model we could confirm that a single ECP cycle is not able to alleviate GvHD. These observations are in concordance with the immunological finding that immune tolerance can be induced by steady-state levels of apoptotic cells [28]. In the conventional ECP setup of our study, GvHD animals were injected with genetically identical 8-MOP/UV-A treated cells from another cohort of B6→BALB/c transplanted GvHD mice. This approach corresponds to the conventional human ECP setup with the major difference that the cells were not obtained from the ECP treated mouse itself. Thus, important evidence arises from our observation that the main mechanism of ECP is not the reduction of alloreactive T-cells, but rather the application of 8-MOP/UV-A treated apoptotic cells in general. Hence, it is maybe not necessary that cells for extracorporeal treatment are from the patient’s origin. Therefore, our initial hypothesis was that cells from another source could perhaps replace the patient’s apheresis procedure to avoid this physical intervention in patients with severe acute GvHD.

In a MHC matched and minor mismatched mouse model for GvHD, Capitini and co-workers found that 8-MOP/UV-A treated splenocytes from healthy non-GvHD mice with the background of BMT donor origin were able to reduce GvHD associated weight loss [29]. We wondered whether this modified ECP setup in which ECP cells are not patient-derived is able to reduce GvHD clinical score or even enhance the survival rate in an MHC mismatched model for acute GvHD. Furthermore we compared the efficacy of this modified ECP setup with our conventional ECP setup described above. Interestingly, we could observe only subtle therapeutic effects performing the modified ECP with 8-MOP/UV-A treated splenocytes from healthy B6 mice representing the genetic background of BMT donor origin. Marginal improvements in terms of clinical symptoms and survival rates were visible in the first 35 days after BMT but did not persistent until the end of the observation time at day 56. Injection of increased numbers of 8-MOP/UV-A treated splenocytes or prolongation of the therapeutic period by performing four instead of three ECP applications led to the same results.

Our findings are consistent with the hypothesis, that alloreactive apoptotic T-cells are essential for the induction of ECP mediated tolerance [30]. Since 8-MOP/UV-A treated cells in our modified ECP setup became apoptotic before getting into contact with the patient’s MHC antigens, the cells were not alloreactive at any time point. Due to the lack of reactivity, these cells were possibly not able to induce tolerance in a sufficient and long-lasting manner. However, since we could observe subtle therapeutic effects of the modified setup with ECP cells derived from healthy B6 mice this approach could perhaps be successful in other settings e.g. in chronic GvHD. Notably, we could not observe any increase of GvHD score or mortality after injection of allogeneic 8-MOP/UV-A treated leukocytes. This is a crucial point because no additional alloreactions in the GvHD patient would be tolerable. In concordance with the well known effect of apoptosis induction by ECP treatment [15], [31], [32], we suggest that standard 8-MOP/UV-A treatment of allogeneic donor leukocytes is sufficient to prevent alloreactions of these cells in the recipient.

Since we could not find any negative effects of ECP with allogeneic 8-MOP/UV-A treated cells, the concept of replacing patient’s cells by different types of allogeneic cells from healthy donors for the treatment of acute and chronic GvHD should be investigated in future studies.

## Supporting Information

Table S1 **ARRIVE Guidelines Checklist.** Overview and background information about the *in vivo* experiments according to the ARRIVE Guidelines.
(PDF)
